# Association Between Sarcopenia Defined by the Asian Working Group for Sarcopenia 2025 Criteria and Cognitive Function in Middle‐Aged Community‐Dwelling Adults

**DOI:** 10.1111/ggi.70426

**Published:** 2026-02-24

**Authors:** Daijo Shiratsuchi, Hyuma Makizako, Kento Tabira, Yuto Miyake, Takuro Kubozono, Mitsuru Ohishi

**Affiliations:** ^1^ Department of Physical Therapy, School of Health Sciences, Faculty of Medicine Kagoshima University Kagoshima Japan; ^2^ Graduate School of Health Sciences Kagoshima University Kagoshima Japan; ^3^ Department of Cardiovascular Medicine and Hypertension, Graduate School of Medical and Dental Sciences Kagoshima University Kagoshima Japan

Dear Editor,

Sarcopenia, an age‐related condition involving a progressive reduction in skeletal muscle mass and strength, is associated with an increased risk of disability and mortality and a reduced quality of life [1]. In older adults, growing evidence links sarcopenia to poorer cognitive performance, including mild cognitive impairment and dementia [2]. Although sarcopenia has traditionally been studied in adults aged 65 years and older, recent consensus statements indicate that muscle deterioration can already be evident in middle age [3]. In midlife, subtle inter‐individual differences in cognitive performance may reflect early pathophysiological processes linking muscle and brain health [4]. The updated Asian Working Group for Sarcopenia (AWGS2025) criteria recently proposed reference values for assessing muscle health in middle‐aged individuals aged 50–64 years [3]. However, evidence on how sarcopenia, defined using these updated criteria, relates to cognitive performance in community‐dwelling adults aged 50–64 years remains limited. To address this gap, we examined the cross‐sectional association between the AWGS2025‐defined sarcopenia and cognitive function in middle‐aged community‐dwelling adults in Japan.

This cross‐sectional analysis used data from the Tarumizu Study 2023–2024, an ongoing community‐based health study in Tarumizu City, Kagoshima, Japan [5]. Among 222 residents aged 50–64 years who participated in community health examinations, individuals with a history of stroke (*n* = 5), unavailable handgrip strength due to severe hypertension (*n* = 7), or inability to complete the Virtual Reality‐Based Cognitive Function Examination (VR‐E) because of visual conditions (*n* = 8) were excluded. This study was approved by the Ethics Committee on Epidemiological and Related Studies of Sakuragaoka Campus, Kagoshima University (approval number: 170351). Appendicular skeletal muscle mass (ASM) was assessed using a multi‐frequency bioelectrical impedance analyzer (MC‐780A‐N; TANITA Co. Ltd., Tokyo, Japan), which provided whole‐body and segmental body composition data [6]. Handgrip strength was measured as the maximum value obtained from the dominant hand using a Smedley‐type handheld dynamometer (Grip‐D; Takei Ltd., Niigata, Japan) [7]. Sarcopenia was defined as low muscle mass (height‐adjusted and/or BMI‐adjusted) and low handgrip strength [3]. Cognitive function was assessed using the VR‐E, which provides a total score and evaluates five cognitive domains (memory, judgment, spatial cognition, calculation, and language) [8]. Multiple linear regression models were adjusted for age, sex, number of medications, hypertension, and education.

The prevalence of sarcopenia was 9.4% (19/202), with rates of 12.9% in males (11/85) and 6.8% in females (8/117) (Figure 1a). The median age of the participants was 57.0 years (interquartile range, 54.0–61.0), with no significant difference between the sarcopenia and non‐sarcopenia groups. The number of medications was greater in the sarcopenia group than in the non‐sarcopenia group. Individuals with sarcopenia had lower handgrip strength and BMI‐adjusted ASM than those without sarcopenia, whereas height‐adjusted ASM was similar between groups (Table S1).

**FIGURE 1:**
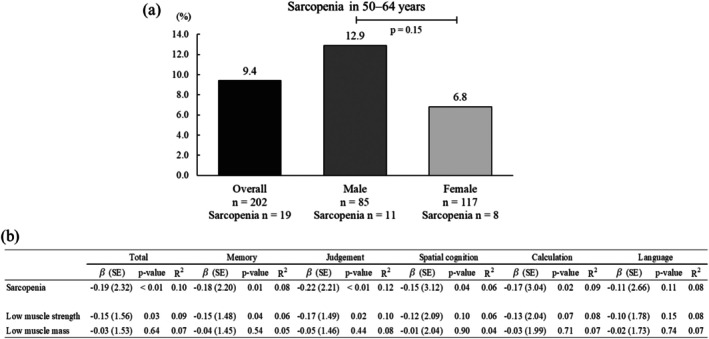
Prevalence of sarcopenia and its associations with cognitive function. (a) Prevalence of sarcopenia among community‐dwelling adults aged 50–64 years, defined according to the AWGS2025 criteria. Sex differences in prevalence were examined using Fisher's exact test. (b) Associations between sarcopenia and cognitive function based on multiple linear regression models. Each row represents a separate regression model adjusted for age, sex, number of medications, hypertension, and education. *β* = standardized coefficient; SE = standard error; VR‐E = virtual reality‐based cognitive function examination.

In fully adjusted models, participants with sarcopenia showed lower cognitive performance than those without sarcopenia. Sarcopenia was negatively associated with the total VR‐E score (*β* = −0.19, *p* < 0.01), memory (*β* = −0.18, *p* = 0.01), judgment (*β* = −0.22, *p* < 0.01), spatial cognition (*β* = −0.15, *p* = 0.04), and calculation (*β* = −0.17, *p* = 0.02). No significant association was observed for language (*β* = −0.11, *p* = 0.11). The adjusted models explained 6%–12% of the variance in cognitive outcomes (Figure 1b).

Sarcopenia can be present in midlife, with prior meta‐analytic data indicating that 8%–36% of adults younger than 60 years meet the diagnostic criteria [9]. These estimates are largely based on applying definitions developed for older adults to younger populations, contributing to substantial heterogeneity. The AWGS2025 criteria, which provide reference values for middle‐aged individuals, are expected to improve comparability. We showed that sarcopenia, as defined by the AWGS2025 criteria, is associated with lower cognitive function among middle‐aged community‐dwelling adults. The inclusion of BMI‐adjusted ASM in the updated criteria may help identify aspects of muscle health that are not fully captured by height‐adjusted measures alone, particularly in midlife. However, given the small number of patients with sarcopenia and the cross‐sectional nature of the data, the observed associations should be interpreted cautiously. There is an association between sarcopenia and poor performance in several cognitive domains, including memory and executive function, and a higher risk of cognitive impairment [10]. In the present study, low muscle strength was associated with lower total and domain‐specific cognitive performance, whereas low muscle mass alone was not. While their coexistence, reflected by sarcopenia, showed more consistent associations across cognitive domains. This suggests that declines in muscle strength and mass may be related to multidomain cognitive changes across the aging process. However, evidence in midlife remains limited, and cognitive differences are generally subtle. In this context, the lower ASM/BMI observed in the sarcopenia group suggests a higher prevalence of individuals with relatively greater adiposity and reduced muscle mass, consistent with a sarcopenic obesity profile. Obesity‐related metabolic and inflammatory pathways have been proposed as shared mechanisms linking muscle dysfunction and cognitive decline [11, 12]. The domain‐spanning associations observed in this study may reflect early variations in cognitive performance related to muscle health; however, these findings should be interpreted cautiously. Because of the small number of participants with sarcopenia, sex‐specific associations could not be examined and should be addressed in future studies. The limitations of this study include its cross‐sectional design, modest sample size, and limited generalizability. Additional research, particularly longitudinal studies, is required to clarify the relationship between midlife sarcopenia and cognitive function.

In summary, AWGS2025‐defined sarcopenia was associated with lower cognitive performance in adults aged 50–64 years. These findings provide descriptive evidence for muscle health and cognition during midlife.

## Author Contributions

D.S.: conceptualization, data curation, formal analysis, investigation, visualization, and writing – original draft. H.M.: conceptualization, data curation, investigation, project administration, resources, supervision, writing – review and editing, and funding acquisition. K.T.: data curation, investigation, and writing – review and editing. Y.M.: data curation, investigation, and writing – review and editing. T.K.: data curation, investigation, and writing – review and editing. M.O.: data curation, investigation, project administration, resources, supervision, and writing – review and editing.

## Funding

This study was supported by research funding from K.K. FOVE. K.K. FOVE played no role in the study design, data analysis, decision to publish, or manuscript preparation.

## Ethics Statement

This study was conducted in accordance with the Declaration of Helsinki and approved by the Institutional Review Board of the Ethics Committee on Epidemiological and Related Studies, Sakuragaoka Campus, Kagoshima University (approval no. 170351).

## Consent

Informed consent was obtained from all participants prior to their involvement in this study. Written informed consent was also obtained from all participants for the publication of their data‐related findings.

## Conflicts of Interest

This study was conducted as a collaborative research project between Towa Pharmaceutical Co. Ltd., K.K., FOVE, and Kagoshima University. Research funding from K.K. FOVE was provided to Kagoshima University and administered by H.M. The other authors declare no conflicts of interest.

## Supporting information


**Table S1:** Participant characteristics by sarcopenia status.

## Data Availability

Research data are not shared.
