# Supplementary material for: Association Between Sarcopenia Defined by the Asian Working Group for Sarcopenia 2025 Criteria and Cognitive Function in Middle‐Aged Community‐Dwelling Adults
Source: Geriatr Gerontol Int. 2026 Feb 24;26(3):e70426. doi: 10.1111/ggi.70426 (PMC12932731; doi:10.1111/ggi.70426)
Supplement: Supplementary file 1 — Table S1: Participant characteristics by sarcopenia status. [file GGI-26-0-s001.docx]

**Table S1. Participant characteristics by sarcopenia status**

| Variables | Overall  (n = 202) | Non-sarcopenia  (n = 183, 90.6%) | Sarcopenia  (n = 19, 9.4%) | p-value |
| --- | --- | --- | --- | --- |
| Age, years | 57.0 (54.0–61.0) | 57.0 (54.0–61.0) | 57.0 (54.0–61.0) | 0.61 |
| Sex, Female, n (%) | 117 (57.9) | 109 (59.6) | 8 (42.1) | 0.15 |
| Height, cm | 161.1 (154.6–168.2) | 161.0 (155.0–168.7) | 161.6 (147.2–166.3) | 0.04 |
| Body weight, kg | 61.2 (52.6–71.1) | 61.1 (52.5–71.0) | 64.0 (53.7–72.9) | 0.96 |
| BMI, kg/m^2^ | 23.4 (21.2–25.8) | 23.2 (21.1–25.7) | 25.1 (22.4–28.5) | 0.11 |
| Medications, number | 1.0 (0.0–2.0) | 1.0 (0.0–2.0) | 2.0 (0.0–4.0) | 0.04 |
| Chronic diseases, n (%) |  |  |  |  |
| Hypertension | 64 (31.7) | 54 (29.5) | 10 (52.6) | 0.07 |
| Hyperlipidemia | 41 (20.3) | 40 (21.9) | 1 (5.3) | 0.13 |
| Diabetes mellitus | 17 (8.4) | 17 (9.3) | 0 (0.0) | 0.38 |
| Education, high school or below, n (%) | 89 (44.1) | 80 (43.7) | 9 (47.4) | 0.81 |
| Living alone, n (%) | 25 (12.4) | 22 (12.0) | 3 (15.8) | 0.71 |
| Muscle strength |  |  |  |  |
| Handgrip, kg | 27.1 (21.4–36.9) | 27.6 (22.7–37.9) | 23.9 (17.9–30.0) | < 0.01 |
| Low muscle strength, n (%) | 51 (25.2) | 32 (17.5) | 19 (100.0) | < 0.01 |
| Muscle mass |  |  |  |  |
| ASM/height^2^ | 7.1 (6.5–8.3) | 7.1 (6.5–8.3) | 7.0 (6.2–8.2) | 0.44 |
| ASM/BMI | 0.81 (0.69–0.96) | 0.82 (0.69–0.97) | 0.68 (0.54–0.89) | 0.01 |
| Low muscle mass, n (%) | 58 (28.7) | 39 (21.3) | 19 (100.0) | < 0.01 |
| VR-E, scores |  |  |  |  |
| Total | 96.0 (93.0–98.0) | 96.0 (93.0–98.0) | 96.0 (85.0–97.0) | 0.42 |
| Memory | 97.0 (95.0–98.0) | 97.0 (95.0–98.0) | 97.0 (77.0–98.0) | 0.36 |
| Judgement | 96.0 (92.0–98.0) | 96.0 (93.0–98.0) | 96.0 (89.0–97.0) | 0.24 |
| Spatial cognition | 95.0 (89.0–98.0) | 95.0 (89.0–98.0) | 96.0 (86.0–98.0) | 0.63 |
| Calculation | 95.0 (90.0–98.0) | 95.0 (90.0–99.0) | 94.0 (86.0–98.0) | 0.29 |
| Language | 99.0 (96.0–100.0) | 99.0 (96.0–100.0) | 99.0 (94.0–100.0) | 0.97 |

Low muscle strength was defined as handgrip strength <34 kg in males and <20 kg in females.

Low muscle mass was defined using BIA-based cutoffs (ASM/height² <7.6 kg/m² in males and <5.7 kg/m² in females, or ASM/BMI <0.90 in males and <0.63 in females).

Abbreviations: ASM = appendicular skeletal muscle mass; IQR = interquartile range; VR-E = virtual reality-based cognitive function examination.

Continuous variables are presented as median (interquartile range) and were compared using the Mann–Whitney U test. Categorical variables were compared using Fisher’s exact test.
